# Vitamin K deficiency, evaluated with higher serum ucOC, was correlated with poor bone status in women

**DOI:** 10.1186/s40101-020-00221-1

**Published:** 2020-04-10

**Authors:** Natsumi Tanaka, Kazuhiko Arima, Takayuki Nishimura, Yoshihito Tomita, Satoshi Mizukami, Takuhiro Okabe, Yasuyo Abe, Shin-ya Kawashiri, Michiko Uchiyama, Yuzo Honda, Ritsu Tsujimoto, Mitsuo Kanagae, Makoto Osaki, Kiyoshi Aoyagi

**Affiliations:** 1 Department of Public Health, Nagasaki University Graduate School of Biomedical Sciences, 1-12-4 Sakamoto, Nagasaki, 852-8523 Japan; 2 Department of Orthopedic Surgery, Nagasaki University Graduate School of Biomedical Sciences, Nagasaki, Japan; 3 Department of Rehabilitation, Nishi-Isahaya Hospital, Isahaya, Japan; 4 Department of Community Medicine, Nagasaki University Graduate School of Biomedical Sciences, Nagasaki, Japan

**Keywords:** Alcohol drinking, Body mass index, Bone health, Current smoking, Sexes, Vitamin K

## Abstract

**Background:**

An increase in serum undercarboxylated osteocalcin concentrations suggests vitamin K deficiency. Clinical intervention studies suggested that the vitamin K supplementation might contribute to preventing bone loss in postmenopausal women. Evidence on the relationship between serum undercarboxylated osteocalcin (ucOC) levels and bone parameters of quantitative ultrasound (QUS) is limited. We examined the correlation between serum ucOC concentrations and bone status as measured by QUS among middle-aged and older Japanese men and women.

**Methods:**

The subjects were community-dwelling men (*n* = 358) and women (*n* = 503) aged ≥ 40 years in Japan. Heel QUS parameters, including the stiffness index, speed of sound, and broadband ultrasound attenuation, were measured. Serum ucOC concentrations were measured by electrochemiluminescence immunoassay. Grip strength was measured in the dominant hand. Information on alcohol drinking, current smoking, exercise, and menopause in women was collected.

**Results:**

Serum ucOC concentrations were significantly associated with age in both sexes. Serum ucOC concentrations in men were higher at ≥ 80 years than those in the age groups of 40–49, 50–59, and 60–69 years. Serum ucOC concentrations in women were higher in the age groups of 50–59 and 60–69 years than those at 40–49 years. Partial correlation analysis adjusting for covariates (age, body mass index, grip strength, alcohol drinking, current smoking, and exercise in men; age, body mass index, grip strength, alcohol drinking, current smoking, exercise, and menopause in women) showed that serum ucOC concentrations were negatively significantly correlated with all QUS parameters in women. Serum ucOC concentrations were not correlated with them in men.

**Conclusions:**

Vitamin K deficiency, evaluated with higher serum ucOC, was correlated with poor bone status in women.

## Background

Osteoporosis and associated fragility bone fracture is a major health problem in the elderly. Osteoporosis was defined as a systemic skeletal disease characterized by low bone mass and microarchitectural deterioration of bone tissue, with a consequent increase in bone fragility and susceptibility to fracture. Low bone mass is one of the reliable objective evaluations for osteoporosis and is reported to be associated with various factors, such as gender, age, several foods, and behavior in daily life. Among these factors vitamins were safe and thought to be effective targets for the prevention against osteoporosis.

Vitamin K is a liposoluble vitamin discovered in 1929, and its name derived from the German word koagulation. Clinical intervention studies suggested that the vitamin K supplementation might contribute to preventing bone loss in postmenopausal women [1, 2]*.* Vitamin K can be measured in plasma, but the values alone may not be sufficient to assess the biological vitamin K status because of its many isoforms and different bioavailability. Phylloquinone is an isoform found in human diet and is synthesized by plants and green vegetables. Menaquinones with several subtypes are another isoform of vitamin K in meat and are endogenously synthesized by intestinal bacteria. Food frequency questionnaires can assess the intake of vitamin K, but assessing the activity rather than intake seems clinically more appropriate. Objective measurement by quantifying undercarboxylation of osteocalcin seems to be sensitive for the activity of vitamin K.

Osteocalcin (OC) is a non-collagenous protein that is secreted by osteoblasts, and has three glutamic acid residues [3]. These residues in OC are γ-carboxylated in the presence of vitamin K, and OC becomes carboxylated osteocalcin (Gla-OC), which binds to calcium ions in hydroxyapatite (hydroxylated calcium phosphate). While Gla-OC deposits to bone, at an insufficient vitamin K dietary intake, OC does not undergo complete γ-carboxylation, and undercarboxylated osteocalcin (ucOC) is released [4]. Because ucOC is not taken in by the bone matrix and is released in the blood, an increase in blood ucOC concentrations indicates a lack of vitamin K and a decrease in Gla-OC [4].

Low vitamin K intake is associated with an increased risk of hip fractures [5, 6]. Vergnaud et al. [7] showed that serum ucOC concentrations predict the risk of hip fracture independent of femoral neck bone mineral density (BMD) in older women. Bullo et al. [8] showed that vitamin K intake is associated with a higher BMD in men and women, but Booth et al. [9] showed an association only in women, not in men. Some studies have shown that elevated serum ucOC concentrations are associated with a low BMD in women [10, 11], but other studies did not show this association [12, 13].

Quantitative ultrasound (QUS) provides information that is more useful than just density for estimating bone strength [14]. QUS is even a promising alternative to dual energy X-ray absorptiometry for estimating BMD [15, 16]. Liu et al. [12] showed that serum ucOC concentrations were negatively correlated with QUS parameters in older women. Recently, Suzuki et al. [17] showed a correlation between serum ucOC concentrations and QUS parameters, such as the speed of sound (SOS), in female college students. Therefore, evidence on the relationship between serum ucOC concentrations and QUS parameters is limited in women. Furthermore, to the best of our knowledge, there have been no reports on correlations between serum ucOC concentrations and QUS parameters in men. We examined the correlations between serum ucOC concentrations, as an objective indicator for the vitamin K status, and bone status as measured by QUS in middle-aged and older Japanese men and women.

## Methods

The subjects were community-dwelling men and women aged 40 years and older in Unzen City, Nagasaki Prefecture, Japan. The population aged 40 years and older in this City was approximately 13,000. Subjects were recruited from men and women who participated in periodic health examinations in 2011–2013. A total of 441 men (mean [SD] age 66.1 [9.9], range 40–92 years) and 637 women (mean [SD] age 66.0 [9.2], range 40–89 years) participated in this study. All subjects provided written, informed consent before examinations. This study was approved by the Ethics Committee of Nagasaki University Graduate School of Biomedical Sciences.

Heel QUS parameters, including the stiffness index, SOS, and broadband ultrasound attenuation (BUA), were measured using a Lunar Achilles device (GE Lunar Corp., Madison, WI). Spot blood samples were collected. Serum ucOC concentrations were measured by electrochemiluminescence immunoassay. Height (cm) and weight (kg) were measured with light clothing and without shoes, and the body mass index (BMI) was calculated as weight/height squared (kg/m^2^). Grip strength was measured using a hydraulic hand dynamometer (Jamar hydraulic hand dynamometer 5030 J1; Jafayette Instrument Company, Inc., Jafayette, IN). The average performance from two trials using the participants’ dominant hand was used as the result for analysis. Since lifestyle is important to physical and health status [18–20], information on alcohol drinking (≥ 40 g/day in men, ≥ 20 g/day in women), current smoking (yes/no), exercise (at least 30 min twice per week), and menopause (yes/no only in women) was collected by interview.

Subjects with missing values for any variables were excluded from the analysis, with a total of 358 men and 503 women for the final data analysis. The Student *t* test or the chi-square test was used to examine the differences in variables between sexes. One-way ANOVA was used to compare serum ucOC concentrations among 10-year age groups. As ucOC and QUS parameters were out of normal distribution, Pearson’s product-moment correlations and partial correlations adjusting for age, BMI, grip strength, alcohol drinking, current smoking, exercise, and menopause (for women) were calculated to assess correlations between serum ucOC concentrations and QUS parameters [20–23]. A *p* value of less than 0.05 was considered significant. The data were analyzed using the Statistical Analysis System software package version 9.4 (SAS Institute, Cary, NC).

## Results

Table 1 shows the characteristics of the subjects. QUS parameters (stiffness index, SOS, and BUA) were higher in men than those in women (*p* < 0.001 for these). Serum ucOC concentrations in women were higher than those in men (*p* < 0.001).

**Table 1:** Characteristics of the subjects

	Men (*n* = 358)	Women (*n* = 503)	*p* value
Mean (SD)			
Age	65.9 (9.7)	66.0 (9.0)	0.872
Height (cm)	163.9 (6.7)	151.9 (5.8)	< 0.001
Weight (kg)	63.7 (10.0)	51.4 (8.4)	< 0.001
BMI (kg/m^2^)	23.7 (3.1)	22.2 (3.2)	< 0.001
Stiffness index	86.6 (16.2)	70.3 (14.6)	< 0.001
SOS (m/sec)	1546.1 (29.8)	1525.8 (26.1)	< 0.001
BUA (dB/MHz)	110.7 (13.9)	94.6 (13.4)	< 0.001
ucOC (ng/mL)	3.7 (3.1)	5.5 (3.8)	< 0.001
Grip strength (kg)	37.2 (8.1)	24.2 (5.7)	< 0.001
Number (%)			
Alcohol drinking^a^	21 (5.9)	8 (1.6)	< 0.001
Current smoking	61 (17.0)	8 (1.6)	< 0.001
Exercise	96 (26.8)	154 (30.6)	0.226
Menopause	-	463 (92.1)	-

*BMI* body mass index, *SOS* speed of sound, *BUA* broadband ultrasound attenuation, *ucOC* undercarboxylated osteocalcin

^a^ ≥ 40 g/day in men, ≥20 g/day in women

Table 2 shows the mean (SD) of QUS parameters by age groups. QUS parameters significantly decreased with age in both sexes (*p* < 0.001).

**Table 2:** Mean (SD) of heel quantitative ultrasound parameters by age group

Age group (years)	40–49	50–59	60–69	70–79	80 over	*p* value
Men	(*n* = 30)	(*n* = 46)	(*n* = 140)	(*n* = 124)	(*n* = 18)	
Stiffness index	98.2 (14.6)	89.6 (14.5)	87.6 (15.6)	83.2 (16.7)	76.0 (12.2)	< 0.001
SOS (m/sec)	1569.1 (27.4)	1551.5 (28.3)	1545.8 (29.9)	1541.5 (29.0)	1529.3 (18.7)	< 0.001
BUA (dB/MHz)	118.4 (12.3)	113.0 (12.3)	112.3 (13.3)	107.5 (14.5)	101.8 (11.5)	< 0.001
Women	(*n* = 27)	(*n* = 87)	(*n* = 189)	(*n* = 178)	(*n* = 22)	
Stiffness index	88.3 (14.5)	78.1 (14.4)	71.6 (12.8)	64.0 (12.1)	56.4 (8.5)	< 0.001
SOS (m/sec)	1550.1 (35.7)	1539.6 (24.9)	1527.8 (22.6)	1516.0 (23.0)	1504.6 (16.4)	< 0.001
BUA (dB/MHz)	111.6 (13.0)	100.6 (13.4)	95.8 (12.3)	89.4 (11.1)	82.6 (7.7)	< 0.001

*SOS* speed of sound, *BUA* broadband ultrasound attenuation

Table 3 shows the mean (SD) of serum ucOC concentrations by age groups. Serum ucOC concentrations significantly increased with age in men and women (*p* = 0.018 and *p* = 0.016, respectively). Serum ucOC concentrations in men were higher in the age group of 80 years and older compared with those in the age groups of 40–49, 50–59, and 60–69 years. Serum ucOC concentrations in women were higher in the age groups of 50–59 and 60–69 years than those in the age group of 40–49 years.

**Table 3:** Mean (SD) of undercarboxylated osteocalcin (ucOC) by age groups

Age group (years)	40–49	50–59	60–69	70–79	80 over	*p* value
Men	(*n* = 30)	(*n* = 46)	(*n* = 140)	(*n* = 124)	(*n* = 18)	
ucOC (ng/mL)	3.1 (1.1)	3.1 (1.3)	3.6 (2.6)	3.8 (3.7)	5.9 (6.1)^a^	0.018
Women	(*n* = 27)	(*n* = 87)	(*n* = 189)	(*n* = 178)	(*n* = 22)	
ucOC (ng/mL)	3.5 (1.7)	6.0 (3.4) ^b^	5.9 (4.3)^b^	5.3 (3.7)	4.7 (3.7)	0.016

^a^*p* < 0.05 compared with the age groups of 40–49, 50–59, and 60–69 years

^b^*p* < 0.05 compared with the age group of 40–49 years

Table 4 shows simple and partial correlation coefficients between serum ucOC concentrations and the stiffness index, SOS, and BUA. Simple correlation analysis showed that serum ucOC concentrations were negatively correlated with all QUS parameters in women (*p* = 0.013, 0.044, and 0.015, respectively) and with the stiffness index and SOS in men (*p* = 0.031 and 0.030). Partial correlation analysis adjusted for covariates (age, BMI, grip strength, alcohol drinking, current smoking, and exercise in men; age, BMI, grip strength, alcohol drinking, current smoking, exercise, and menopause in women) showed that serum ucOC concentrations were negatively significantly correlated with all QUS parameters in women (*p* = 0.019, 0.034, and 0.045, respectively). Serum ucOC concentrations were not correlated with the stiffness index, SOS, or BUA in men (*p* = 0.229, 0.149, and 0.421, respectively).

**Table 4:** Simple and partial correlation coefficients between ucOC concentrations and heel quantitative ultrasound parameters

	Men (*n* = 358)		Women (*n* = 503)	
	*r*	*p* value	*r*	*p* value
Simple				
Stiffness index	−0.114	0.031	−0.111	0.013
SOS (m/sec)	−0.115	0.030	−0.090	0.044
BUA (dB/MHz)	−0.097	0.067	−0.109	0.015
Partial^a^				
Stiffness index	−0.064	0.229	−0.105	0.019
SOS (m/sec)	−0.077	0.149	−0.095	0.034
BUA (dB/MHz)	−0.043	0.421	−0.090	0.045

*ucOC* undercarboxylated osteocalcin, *SOS* speed of sound, *BUA* broadband ultrasound attenuation

^a^Adjusted for age, body mass index, grip strength, alcohol drinking (≥40 g/day), current smoking, and exercise in men; age, body mass index, grip strength, alcohol drinking (≥20 g/day), current smoking, exercise, and menopause in women

## Discussion

We showed that after adjusting for covariates, serum ucOC concentrations were negatively significantly correlated with the stiffness index, SOS, and BUA only in women. To the best of our knowledge, this is the first study to examine the correlation between serum ucOC concentrations and QUS parameters in men, in whom no correlations were found.

In the present study, serum ucOC concentrations significantly were associated with age in both sexes. Serum ucOC concentrations were higher after 50 years old in women. Previous studies showed that serum ucOC concentrations decreased with age in men [24, 25] and increased postmenopausally in women [10, 24–26]. These findings are consistent with our findings in women, but not in men. Because vitamin K is the main factor determining the state of carboxylation of OC, an increase in serum ucOC concentrations suggests vitamin K deficiency. There may be variations in vitamin K intake among individuals, which could in part contribute to the inconsistent results in men.

There were significant differences in bone mass between men and women. Also there was gender difference in the concentration of ucOC in community-dwelling subjects living in the same city. Some factors may be associated with the gender difference in activity of vitamin K about bone health, such as the differences in the amount of bone mass, amount of sex hormones, prevalence of drinking and smoking, and postmenopausal hormonal change. Further study is necessary to assess the mechanism of gender difference in the function of vitamin K.

QUS measurements reflect qualitative properties [27], considering that BUA is mainly affected by structural characteristics of trabecular bone, such as porosity [8, 28], and that SOS is an indicator of the elastic properties of bone [29]. Our results suggest that elevated serum ucOC concentrations (vitamin K deficiency) contribute to increased porosity and decreased elasticity.

The intake of environmental resource as a food, including several kinds of vitamins, is one of the physiological adaptations because human cannot synthesize any vitamins in their own cells. It is well established that the deficiency of vitamin K leads to health problems of blood clotting. Not only in the pathological status, but also in physiological status of community-dwelling subjects, vitamin K may play a role for bone health. Further study may discover the different physiological adaptation in absorbance, bioavailability or response to vitamins, which may contribute to adapt to the poor environmental resource.

Vitamin K deficiency is associated with hip or vertebral fractures [5–7, 30]. Liu et al. [12] showed that serum ucOC concentrations were more strongly correlated with calcaneal QUS parameters (e.g., ultrasonic transmitted velocity) than femoral neck BMD. This finding suggests that ucOC content of bone may adversely affect bone strength, with more effect on bone quality than on BMD. Vitamin K deficiency may adversely affect bone quality more than bone mass.

This study has several limitations. First, because the study was cross-sectional in nature, proving causal relationships between ucOC concentrations and QUS parameters was difficult. A longitudinal study is required to determine this causality. Second, because BMD measurements were not available in this study, we could not assess the degree of bone quality (QUS) and mass (BMD). Third, because we could not assess nutritional status, dietary vitamin K levels were unknown. Forth, because we showed an association between ucOC and QUS parameter adjustment for gender, age, body mass index, grip strength, alcohol drinking, current smoking, exercise, and menopause, we could not assess other possible factors influencing bone health, including nutrition and occupations. Fifth, because our subjects were all Japanese who voluntarily participated in periodic health examinations, our results have to be interpreted with caution as the selection bias.

## Conclusion

Vitamin K deficiency, evaluated with higher serum ucOC, was correlated with poor bone status in women, but not in men.

## Data Availability

The datasets used and analyzed during the current study are available from the corresponding author on reasonable request.

## Abbreviations

BMI: Body mass index
BMD: Bone mineral density
BUA: Broadband ultrasound attenuation
OC: Osteocalcin
QUS: Quantitative ultrasound
SOS: Speed of sound
ucOC: Undercarboxylated osteocalcin
